# Vitamin D and Psoriasis Pathology in the Mediterranean Region, Valencia (Spain)

**DOI:** 10.3390/ijerph111212108

**Published:** 2014-11-25

**Authors:** Maria Morales Suárez-Varela, Paloma Reguera-Leal, William B. Grant, Nuria Rubio-López, Agustín Llopis-González

**Affiliations:** 1 Unit of Public Health, Hygiene and Environmental Health, Department of Preventive Medicine and Public Health, Food Science, Toxicology and Legal Medicine, University of Valencia, 46100 Valencia, Spain; E-Mails: palomaregueraleal@hotmail.com (P.R.-L); nrubiolopez@hotmail.com (N.R.-L.); agustin.llopis@uv.es (A.L.-G.); 2 CIBER Epidemiología y Salud Pública (CIBERESP), 28029 Madrid, Spain; 3 Center for Advanced Research in Public Health (CSISP-FISABIO), 46010 Valencia, Spain; 4 Sunlight, Nutrition and Health Research Center, P.O. Box 641603, San Francisco, CA 94164, USA; E-Mail: wbgrant@infionline.net

**Keywords:** vitamin D, psoriasis, Mediterranean region, diet

## Abstract

Vitamin D has important immunomodulatory effects on psoriasis in the Mediterranean region. To measure vitamin D intake in subjects with and without psoriasis, and to find an association with relevant clinical features, a case-control study was performed using cases (n = 50, 50% participation rate) clinically diagnosed with psoriasis and 200 healthy subjects (39.5% participation rate), leaving a final sample of 104 people. A survey was conducted using a food frequency questionnaire and clinical histories. Cases and controls were compared using univariate and multivariate analyses. We observed insufficient intake of cholecalciferol (vitamin D3) or ergocalciferol (vitamin D2) for both cases and controls. Patients with psoriasis were at greater risk of associated pathologies: dyslipidaemia (OR: 3.6, 95% CI: 0.8–15.2); metabolic syndrome (OR: 3.3, 95% CI: 0.2–53.9); hypertension (OR: 1.7, 95% CI: 0.4–7.2). Insufficient vitamin D intake in both psoriasis patients and controls in the Mediterranean population, and cardiovascular comorbility is more frequent in patients with psoriasis.

## 1. Introduction

Psoriasis is a common inflammatory skin disorder with variable morphology, distribution, severity and course [1]. Although the cause of psoriasis remains unknown, increasing evidence suggests that psoriasis is a complex disorder caused by the interaction of multiple genes, the immune system [2] and environmental factors [3]. Although psoriasis occurs worldwide [1,4,5], its prevalence varies between 0.6–4.8% [6]. While the genetic influence on psoriasis is well-established, the role of environmental factors is less well-defined. Overweight and obesity have also been identified as risk factors for psoriasis and/or a flare-up of the disease [7].

There has been much debate as to defining vitamin D insufficiency. Optimal concentration of vitamin D [25(OH)D] for maximum effects should be 30–50 ng/mL (75–125 nmol/L) [8]. It is generally agreed that a serum level of vitamin D [25(OH)D] below 20 ng/mL (or 50 nmol/L) is an indication of vitamin D deficiency [8,9], which has long since been recognised as a pathological condition characterised by muscle weakness, rickets or osteomalacia [9,10]. Vitamin D insufficiency, distinguished as a serum level of 25(OH)D ranging from 10 to 30 ng/mL (25–75 nmol/L) with no overt clinical symptoms, has recently become an important concern [11]. Vitamin D insufficiency is extremely common in Europe and the USA, where its prevalence in the general population is estimated to be as high as 50% [12].

Health authorities have used different cut-offs for their definitions of sufficient and optimal statuses, and defining a level of serum 25(OH)D as low or insufficient depends on the level that is defined as normal [13]. Substantial evidence suggests that vitamin D plays a pivotal role in modulating dendritic cell function and in regulating keratinocytes and T-cell proliferation [9,14,15,16]. Epidemiological data have also confirmed that vitamin D deficiency may be a risk for developing autoimmune disease, [10,14,17] including systemic lupus erythematosus [17], Crohn’s disease [18], autoimmune thyroid disease [10], primary biliary cirrhosis [10] and rheumatoid arthritis [14,19].

In vitamin D (cholecalciferol and ergocalciferol), as 25(OH)D, acts via the Vitamin D Receptor (VDR) present in many tissues, skin being one, keratinocytes present VDR [20]. 25(OH)D displays marked growth inhibitory action and favours keratinocyte maturity. Given this activity, insufficient 25(OH)D could prove to be a risk factor in psoriasis whose fundamental pathogenic mechanism affects the cellular immune system (T lymphocytes), as well as the hyperproliferation and differentiation of keratinocytes and angiogenesis [9,15,16].

When psoriasis appears, it may involve a vicious circle where skin, aggravated by the disease, is less capable of synthesising 25(OH)D which, as is well-known, is synthesised by the action of ultraviolet radiation, leaving increasingly less 25(OH)D in the organism. Therefore, it is advisable to eat foods [20] which provide vitamin D to achieve suitable plasma levels because we have previously seen how 25(OH)D plays a key role in the modulation of dentritic cells, the regulation of keratinocytes and the proliferation of T-cells [15,16], which are altered as is case of autoimmune diseases [9,20].

This study estimates the prevalence of vitamin D deficiency intake in patients with chronic psoriasis and analyses the association of vitamin D intake with clinical features by paying special attention to the role of obesity. 

## 2. Methods

### 2.1. Cases and Controls

The cases studied were persons who stated that they have been clinically diagnosed with psoriasis in the previous 12 months. For each case, four control subjects, matched for age (±1 year) and gender, were randomly selected from among the individuals of the sample who did not state that they had psoriasis, as confirmed by a dermatologist and/or general practitioner. Information was collected by revising clinical histories, face-to-face interviews and conducting an intake questionnaire. Information on smoking and drinking habits, body mass index (BMI) and drug intake was used.

Fifty adults with psoriasis and 200 subjects without psoriasis were recruited. Of these, 25 patients with psoriasis accepted to participate in the study, and 79 controls accepted (39.5% participation rate). All the participants were asked to complete the specific food frequency questionnaire on 3 days, validated by the Universidad Complutense de Madrid. [21] Of the participants, 79 did not present psoriasis (controls) and 25 were patients whose psoriasis was moderate to severe. Psoriasis plaques were diagnosed and evaluated by a specialised medical team. BMI was calculated by weight in kg divided by the square of height in m. A BMI of 18–25 kg/m^2^ indicated normal weight, 25–29.9 overweight, ≥30.0 was obesity and ≥35.0 was morbid obesity [7].

The second part of the study involved studying and comparing the intake of nutrients between cases and controls to consider which diet type is capable of altering psoriasis by focusing on vitamin D intake. Cases and controls were recruited at the population level. The inclusion criterion for both cases and controls was if they wished to participate in the study. The exclusion criteria for both groups were if they did not wish to participate in the study and if they were on a special diet. All the subjects received a written informed consent before the study commenced. They were all seen by a dermatologist and a nutritionist, who collected the demographic, biometrics and health status data, and any other relevant details. The obtained data included age, gender, weight, height, BMI, psoriasis duration, concomitant diseases and medication. Cases were age- and gender-matched for comparison. After the data collection, diet was assessed with version 2.16 of the DIAL programme (January 2012) [22], which converts food into nutrients.

### 2.2. Statistical Analysis

Cases and controls were first compared using univariate analysis. All the continuous variables were revised with normal distribution using the Kolmogorov-Smirnov test. The Mann-Whitney U test was used to compare the quantitative variables, while the X2 and Fisher’s exact tests were utilised to compare the qualitative variables. Odds ratios with 95% confidence intervals (95% CI) were estimated separately for each variable using standard case control methods with unconditional logistic regression models forcing the matching variable into all the models. The variables with *p* > 0.15 in the univariate analysis were considered for the multivariate analysis. The variables included in the final multivariate models were first selected using 2 × 2 analyses by assessing the first-order interaction and by confounding multiplicative models. Finally, a backward step-by-step regression was conducted. 

Data are presented as the mean (± 1SD) or number (and percentage), where appropriate. All the tests were 2-tailed. A *p* value of <0.05 was considered statistically significant. Data were analysed using the SPSS 14.0 for Windows (SPSS Inc, Chicago, IL, USA).

## 3. Results 

**Figure 1:**
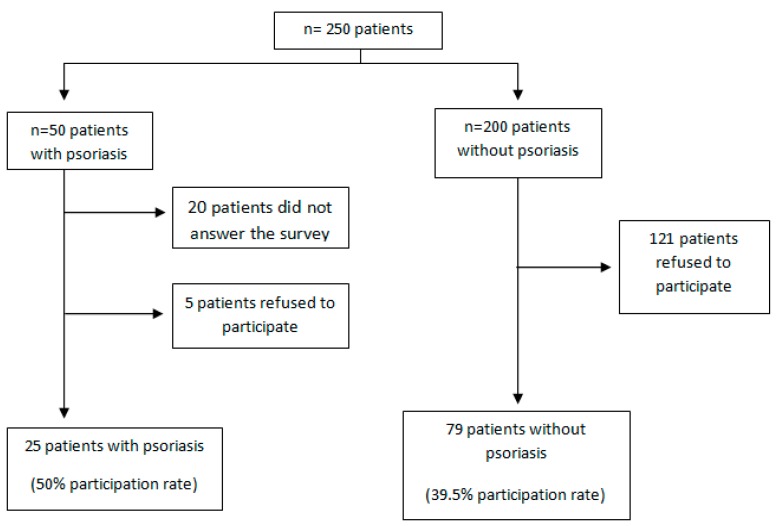
Organizational chart of patients with psoriasis and without psoriasis.

Participation in this study was offered to fifty psoriasis patients. Of these, 20 (40%) did not completely answer the food questionnaire and 5 (10%) did not wish to participate. Thus, the final number of participants was 25 (50% participation rate). Of the 200 healthy subjects who were offered to participate, 121 did not wish to. Therefore, 79 healthy controls (39.5% participation rate) participated, leaving a final sample of 104 people (Figure 1).

Table 1 provides the baseline characteristics and comorbidity of the psoriasis patients (cases) and of the subjects without psoriasis (controls). No statistically significant differences were found between cases and controls as far as age and gender are concerned. However, the weight of the cases with psoriasis was statistically heavier (78 ± 26 kg *vs.* 64 ± 20 kg) (*p* = 0.01). When evaluating BMI, a greater interval for overweight and obesity was maintained among the psoriasis cases (overweight 40%; obesity 36%), implying a risk of 3.39 of being overweight (95% CI: 1.1–10.8) and of 6.3 of being obese (95% CI: 1.8–21.3) among cases. For comorbidity, there were more pathologies found among the cases, of which dyslipidaemia (16%), hypertension (12%) and bone disease (8%) stand out.

**Table 1:** Baseline characteristics and comorbidity of subjects with and without psoriasis.

Baseline Characteristics and Comorbidity	Cases (n = 25)	Controls (n = 79)	*p* Value	Odds Ratio (95% CI)
	Fr (%)/Mean (SD)	Fr (%)/Mean (SD)		
Male gender	11 (44)	32 (40)	0.700	
Mean age ± SD, years	38 ± 15	42 ± 16	0.200	
Mean weight ± SD, kg	78 ± 26	64 ± 2	0.010	
Mean BMI ± SD	27 ± 8	23 ± 8	0.010	
Normal weight	6 (24)	46 (58)	-	1
Overweight	10 (40)	22 (28)	0.004	3.5 [1.1–10.8]
Obesity	9 (36)	11 (14)	0.003	6.3 [1.8–21.3]
Hypertension	3 (12)	6 (8)	0.004	1.6 [0.4–7.2 ]
Dyslipidaemia	4 (16)	4 (5)	0.001	3.6 [0.8–15.5]
Diabetes	1 (4)	3 (4)	0.700	1.1 [0.10–10.6]
Metabolic syndrome	1 (4)	1 (1)	0.001	3.2 [0.2–53.9]
Bone pathology	2 (8)	2 (2)	0.001	1.6 [0.3–9.5]

Notes: Fr: Frequency; SD: Standard Deviation; BMI: Body Mass Index; *p* value <0.05: was considered statistically significant, 95% CI, Confidence Interval.

Table 2 shows that no significant differences in either total macronutrients intake or level of carbohydrates, lipids, proteins and water was found between cases and controls. No bone diseases of genetic origin were taken into account. There were no differences in intake between both groups. The total energy intake was 1930 ± 704 kcal, carbohydrates 190 ± 72 g, lipids 84 ± 33 g, protein 84 ± 33 g and water 2055 ± 807 mL.

**Table 2:** Macronutrient intake in subjects with and without psoriasis.

Macronutrient	Cases (*n* = 25)	Controls (*n* = 79)	Total (*n* = 104)	*p* Value
	Mean ± SD	Mean ± SD	Mean ± SD	
Energy (kilocalories)	1900 ± 1000	1940 ± 570	1930 ± 704	0.700
Carbohydrates (grams)	190 ± 100	190 ± 27	190 ± 72	0.800
Lipids (grams)	78 ± 41	85 ± 30	84 ± 33	0.300
Proteins (grams)	78 ± 41	86 ± 27	84 ± 33	0.400
Water (mL)	190 ± 1200	2100 ± 645	2055 ± 807	0.400

Notes: SD: Standard Deviation; *p* value < 0.05: was considered statistically significant.

Nevertheless, Table 3 reveals that both study groups consumed less vitamin D than the recommended amount. Psoriasis cases’ vitamin D intake is 230 ± 190 UI/day (*p* = 0.03) and the recommended one is 620 ± 330 UI/day. The controls’ vitamin D intake is 290 ± 280 UI/day (*p* = 0.001) and the recommended one 290 ± 280 UI/day.

**Table 3:** Vitamin D intake in subjects with and without psoriasis.

Intake	Cases	R_Cases	*p* Value		Controls	R_Controls	*p* Value
Vitamin D (UI/day)	230 ± 190	620 ± 330	0.030		290 ± 280	540 ± 220	0.001

Notes: R: recommended intake calculated by the DIAL programme; *p* value < 0.05: was considered statistically significant.

## 4. Discussion

In this study intake vitamin D were significantly lower in both groups regarding the recommendation, being higher in patients with psoriasis, which might account for the greater comorbidity relating to these patients’ insufficient vitamin D intake.

The main vitamin D source is cutaneous synthesis via skin through UV radiation. There is some controversy as to exposure doses to UV radiation for skin cancer to appear. Therefore, additional vitamin D intake is necessary, preferably in the form of certain foods, or otherwise as diet supplements [23]. Since sun exposure is necessary for vitamin D synthesis, the benefit/risk ratio that this implies has to be taken into account since today’s lifestyle tends to involve minimum yet habitual sun exposure, which intensifies during the holiday period, and implies a risk of developing skin cancer [16,24]. Thus moderate sun exposure all year round, along with a varied, healthy diet, should be a recommended practice to achieve adequate 25(OH)D levels in the blood. Unfortunately, very few foods contain 25(OH)D and many of them are not eaten regularly [24], which is one of the main reasons why the study population does not consume adequate quantities of vitamin D.

The relation between 25(OH)D and psoriasis has been studied since the 1930s. In 1985, Morimoto *et al.* [25] made a chance discovery; vitamin D3 administration improved psoriasis in isolated cases. The attempts made to employ oral 25(OH)D have been limited by its capacity to alter the calcium metabolism. Analogues of 25(OH)D present poorer hypercalcaemic activity to, for instance, Calcipotriol and tacalcitol and their biological actions, which include regulation of epidermal cell proliferation and differentiation, inhibition of angiogenesis and modulation of cytokines production [26]. Morimoto *et al*. [27] detected less circulating vitamin D3 in subjects with severe psoriasis, this relationship can be partially explained by the liposolubility of vitamin D and its reduced bioavailability in bodies with a high fat content [28]. This may be the reason why our psoriasis patients present more comorbidity as a higher prevalence of overweight or dyslipidaemia. Some research groups have centered their studies on vitamin D receptors. Okita *et al.* [29] studied the polymorphisms of VDR in psoriasis patients, they discovered a significant relation between genotype AA and liver failure in some patients. This finding suggests that 25(OH)D acts as regulator of the metabolic syndrome expression and dyslipidaemia, which accompanies psoriasis, as observed in the present study. 

The type 1 diabetes mellitus is a frequent comorbidity in psoriasis patients. Seasonal variations have been found in the peaks of diabetes mellitus (DM) incidence, which have been associated with periodic oscillations in vitamin D levels [24]. In a large multicentre prospective 4-year study conducted in 51 regions worldwide, an inverse relation between UV radiation (UV-B) in all 51 regions and the incidence of type 1 DM has been verified [30]. Some studies found that vitamin D administration during infancy (2000 UI/day of 25(OH)D) with a follow-up lasting up to 30 years has been reported to significantly reduce the development of type 1 DM [31,32].

Arterial hypertension is another of the pathologies found more frequently among psoriasis patients and is regulated by 25(OH)D through Renin-Angiotensin System inhibition [33]. This relation has been supported by experimental studies, like that of Li *et al*. [34], who verified how administering 1,25-hydroxy vitamin D [1,25(OH)2D] inhibits the gene expression of renin in knockout mice for the expression of the 25(OH)D receptor. Likewise hypertension, which these mice generate spontaneously, can be reverted with both captopril and [1,25(OH)2D] [24,33] and UVA irradiation of human skin caused a significant drop in blood pressure even at moderate UVA doses [35].

The role that 25(OH)D plays in bone pathologies by regulating calcium in the blood to avoid hypocalcaemia and to stimulate mineralisationis well-known. 25(OH)D is able to stimulate the proteins involved in calcium absorption in the intestine, yet when calcium food intake is lacking, the mobilisation of its reserves from bone mass is favoured, which stimulates osteoclastogenesis. Furthermore, 25(OH)D acts with the parathyroid hormone to stimulate calcium re-absorption at the kidney tubules level [24].

Other studies also report the association with obesity found in the present study [7]. Nonetheless, these studies do not report a significant difference in calorie intake, but in BMI, which is greater in psoriasis patients. This led us to consider that there may be differences in their metabolism. Obesity is associated with basic systemic inflammation, characterised by an increase in pro-inflammatory markers such as TNF-α and IL-6 [36]. Adipokines are also dysregulated, which might be the basis of vascular diseases [37], and of insulin resistance and subsequent DM. 

It is necessary to bear in mind that not only there is necessary the ingestion of rich food in vitamin D, but also the development of the vitamin D with the solar exposition exposure, as shown by the studies of the Dead Sea [38] with the normal incidence solar UVB radiation [39]. The quantity of 25(OH)D is very important for general population and psoriasis population, because this group display an altered metabolism [8]. Metabolic syndrome [10,40] (diabetes, hypertension, dyslipidemia, being overweight and obesity) is related with 25(OH)D. Thus evaluating its optimum levels in blood could prevent less comorbidities from appearing.

In short, there is recommended an increase of the vitamin D intake in general in the Mediterranean population and exposure to UV radiation, especially in the patients with psoriasis [8], with food rich in vitamin D such as blue fish (tuna, mackerel, salmon), fish liver, egg yolks and cheese [20]; also enriched food such as milk fortified with vitamin D or the use of vitamin D supplements. In addition new studies that determine both the intake and blood levels of 25(OH)D in psoriasis patients are required.

## 5. Conclusions 

Although more research is need, the considerably low intake of vitamin D in both psoriasis patients and control group indicate the need for proper evaluation of vitamin D status in the Mediterranean population. Further, we identify more cardiovascular comorbililty in psoriasis patients.
